# Editorial: The intersection of cognitive, motor, and sensory processing in aging: links to functional outcomes, volume II

**DOI:** 10.3389/fnagi.2023.1340547

**Published:** 2024-01-04

**Authors:** Uros Marusic, Jeannette R. Mahoney

**Affiliations:** ^1^Institute for Kinesiology Research, Science and Research Centre Koper, Koper, Slovenia; ^2^Department of Health Sciences, Alma Mater Europaea - ECM, Maribor, Slovenia; ^3^Division of Cognitive and Motor Aging, Albert Einstein College of Medicine, Bronx, NY, United States

**Keywords:** sensory processing, cognition, motor performance, aging, multisensory integration

The cognitive processes of encoding, decoding, and interpreting information about biologically significant events represent fundamental neural functions that require extensive integrated neural networks. These processes have played a central role in the course of evolution, giving rise to a variety of specialized sensory organs, each intricately connected to multiple specialized brain regions (Stein and Stanford, 2008). While the sophisticated interactions between neural circuits are of great scientific interest, it is the practical manifestation of these processes that allows us to monitor and understand the physical execution of activities of daily living (ADL). Whether it is the rhythmic act of walking, the successful balancing act needed to avoid falling, the efficient performance of daily activities needed to bathe or eat, or the complex cognitive-motor interplay involved in activities such as dancing, our ability to engage in such structured multisensory endeavors underscores the importance of these neural functions in our daily lives.

Multisensory integration (MSI) is a multimodal process in which the brain combines and coordinates information from multiple sensory modalities such as vision, hearing, touch, and proprioception to produce a unified and coherent perceptual experience. This integration enhances our understanding of the external world, promotes more accurate and reliable perception, and enables effective responses to the environment (Stein et al., 2014). These processes, which are evident at both neural and behavioral levels, can lead to enhancement or attenuation of responses (Wallace et al., 1998; Stein et al., 2009) and significantly influence our sensations, perceptions, and associated behaviors. Response enhancement, which often affects the accuracy and speed of stimulus detection, localization, and identification (Hughes et al., 1994; Ernst and Banks, 2002; Foxe and Schroeder, 2005; Hecht et al., 2008), serves as a reliable index of MSI, which involves a wide range of computations that combine information from multiple sensory modalities.

A well-documented phenomenon in aging is the gradual decline of individual sensory modalities and body functions. These age-related changes affect several areas, including visual acuity (Faubert, 2002; Schieber, 2006), auditory abilities (Van Eyken et al., 2007; Murphy et al., 2018), muscle strength (Hortobágyi et al., 1995; Lindle et al., 1997), and postural balance (Laughton et al., 2003; Marusic et al., 2019), among others. However, the extent to which changes in MSI contribute to age-related deterioration in ADLs remains a less explored area of investigation in the existing literature (de Dieuleveult et al., 2017).

Mahoney et al. have generated evidence for robust, but differential MSI effects in healthy aging and discovered significant links with clinically meaningful outcomes (Mahoney et al., 2014, 2015, 2019; Mahoney and Verghese, 2018, 2020). Specifically, they report that older adults with intact levels of visual-somatosensory integration demonstrate better balance, faster gait velocity and lower incidence of falls, compared to those with integrative deficits. Further they reveal that older adults with MCI and dementia demonstrate significantly reduced magnitude of multisensory integration compared to older adults without cognitive impairments.

The current Research Topic of Frontiers in Aging Neuroscience represents a continuation of Volume I entitled “*The intersection of cognitive, motor, and sensory processing in aging: links to functional outcomes*.” This latest Research Topic includes ten manuscripts that collectively address various facets of sensory integration along with cognitive and motor performance in the context of aging. The primary goal of this Research Topic is to foster new scientific discoveries detailing the complex inter-relationships between sensory, motor, and cognitive functions in aging. Contributors to this Research Topic examine age-related changes in one or more of these systems—sensory, motor, and cognitive—and discuss the impact of these interactions on important functional outcomes, including but not limited to clinical and social aspects. A better understanding of the effective (or ineffective) convergence of these systems holds promise for the wellbeing of older people and offers insights for improving and adapting multimodal interventions aimed at preventing decline and minimizing disability.

Handling et al. and Thompson et al. both focus on predictors and interactions related to cognitive and physical decline in older adults. Handling et al. identify risk factors for dual decline, with depressive symptoms and APOE-ε4 status increasing the odds of developing cognitive and physical decline. In contrast, Thompson et al. employed canonical correlation analysis, unveiling two interconnected clusters of cognitive and physical function tasks in a cross-sectional cohort of cognitively intact older adults. These findings underscore a predominant emphasis on speed-related tasks in both gait and cognition, along with a secondary focus on complex motor and cognitive tests.

Basharat et al. and Šlosar et al. investigate the impact of multisensory processing and virtual reality (VR). Basharat et al. reveal that immersive VR can enhance multisensory processing and improve performance in untrained cognitive tasks. While Šlosar et al. explore the potential of enriched VR environments in mitigating the effects of prolonged bed rest, offering a novel approach to improving rehabilitation outcomes.

In a mini review Meulenberg et al. discuss the potential of dance therapy as a non-pharmacological intervention for Parkinson's disease. Dance interventions induce neuroplastic changes, improving both motor and cognitive functions in PD patients. The authors conclude that more research is needed to determine the optimal dance style and duration for therapeutic benefit.

Tabei et al. investigate the impact of an online physical exercise program with music on cognitive function, particularly working memory in older adults. Their results show significant improvements in working memory, suggesting the potential of online exercise programs to enhance cognitive functions.

In a study protocol, Mahoney et al. outline the potential use of visual-somatosensory integration as a marker for Alzheimer's disease. This protocol details the methodologies used to examine the interplay of sensory, cognitive, and motor functions, as well as study their impact on mobility decline in aging. The main objective is to assess the validity of MSI as a novel non-cognitive, non-invasive, behavioral marker of preclinical Alzheimer's disease.

In a cross-sectional study, Hu et al. investigated age-related changes in cortical control of standing balance and their effects on falls in older adults. Despite some limitations in the reliability of the mechanical perturbation, the results suggest increased cortical recruitment for postural control in older adults and emphasize the need for further studies to improve the understanding of these mechanisms.

Fatokun et al. investigated the relationship between dual-task gait cost (DTC) and white matter hyperintensities (WMH) in Lewy body disorders. Higher DTC was associated with greater frontal WMH burden, providing insights into cognitive-motor interactions in Parkinson's disease and dementia with Lewy bodies.

Finally, Torre et al. investigated the effects of bimanual coordination training on inhibitory functions in older adults. The training, which involved maintenance of an antiphasic pattern and inhibition of the in-phase pattern, effectively delayed the frequency of spontaneous transitions and transferred the benefits to untrained tasks involving inhibitory functions.

Overall, this compilation collectively contributes to our understanding of the complex relationships among sensory, motor, and cognitive functions in the context of aging while shedding light on predictors, interventions, and novel markers that have the potential to improve the wellbeing of older adults. This Research Topic serves as a continuation of ongoing research on the intersection of these functions and highlights the importance of a much-needed multifaceted approach to addressing age-related decline across multiple domains. The research presented here underscores the ongoing commitment to improving the quality of life of older adults and emphasizes the importance of multidisciplinary research in the field of aging neuroscience.

## Author contributions

UM: Writing—original draft, Writing—review & editing. JM: Writing—original draft, Writing—review & editing.
